# An all-in-one UniSam vector system for efficient gene activation

**DOI:** 10.1038/s41598-017-06468-6

**Published:** 2017-07-25

**Authors:** Antonella Fidanza, Martha Lopez-Yrigoyen, Nicola Romanò, Rhiannon Jones, A. Helen Taylor, Lesley M. Forrester

**Affiliations:** 1 Centre for Regenerative Medicine, University of Edinburgh, Edinburgh, UK; 2 Centre for Integrative Physiology, University of Edinburgh, Edinburgh, UK

## Abstract

We have generated a drug-free, all-in-one dCAS9-SAM vector that can activate endogenous gene expression with the potential to modify cell fate. We demonstrate that this strategy can be used in a number of cell lines and avoids exceptionally high levels of gene expression that are observed in standard transgenic approaches. Compared to the multi-plasmid system, this all-in-one vector activates gene expression to a comparable level but the reduced overall DNA content results in significantly higher viability of transfected cells. This allowed us to use the RUNX1C-GFP human embryonic stem cell reporter cell line to monitor gene activation in individual cells and to show that activation could occur at all stages of the cell cycle.

## Introduction

Manipulation of transcription factor expression has been used to programme cell fate and this approach has primarily involved the delivery of exogenous cDNA by plasmid or viral expression vectors. The CRISPR-CAS9 system has revolutionized genome editing^1^ but more recently the catalytically dead CAS9 (dCAS9) system has been used to modulate endogenous gene expression via activation, repression and chromatin modification^2–6^. This strategy has been successfully used to program cell fate^7, 8^. Engineered versions of dCas9 fused to activation domains such as VP64, VPR, p65 or p300 can activate the expression of endogenous genes when directed to their regulatory regions by specific guide RNAs (gRNAs)^3^. Significant progress has been made in the search for the best combinatorial and synergistic approach to mediate endogenous gene expression and the Synergistic Activators Mediators (SAM) is one of the most powerful tool to date^9, 10^. This system combines the use of CAS9-VP64 and a specifically designed multi-domain activator (MS2-p65-HSF1) that binds to MS2 hairpins of engineered gRNAs (gRNA 2.0). The system is based on a multiple plasmid approach and consequently, the need for multiple drug selection to achieve homogeneity. Although a significant advance, these limitations make it challenging to use in cells that are sensitive to viral transduction and/or drug selection. We describe the design and generation of a novel all-in-one strategy that can activate gene expression without drug selection in a number of cell lines including human Embryonic Stem Cells (hESCs). In a proof of principle experiment we demonstrate that the all-in-one vector containing a single gRNA directed to *MyoD1* is able to mediate the trans-differentiation of mouse embryonic fibroblasts into myocytes.

## Results and Discussion

**Figure 1:**
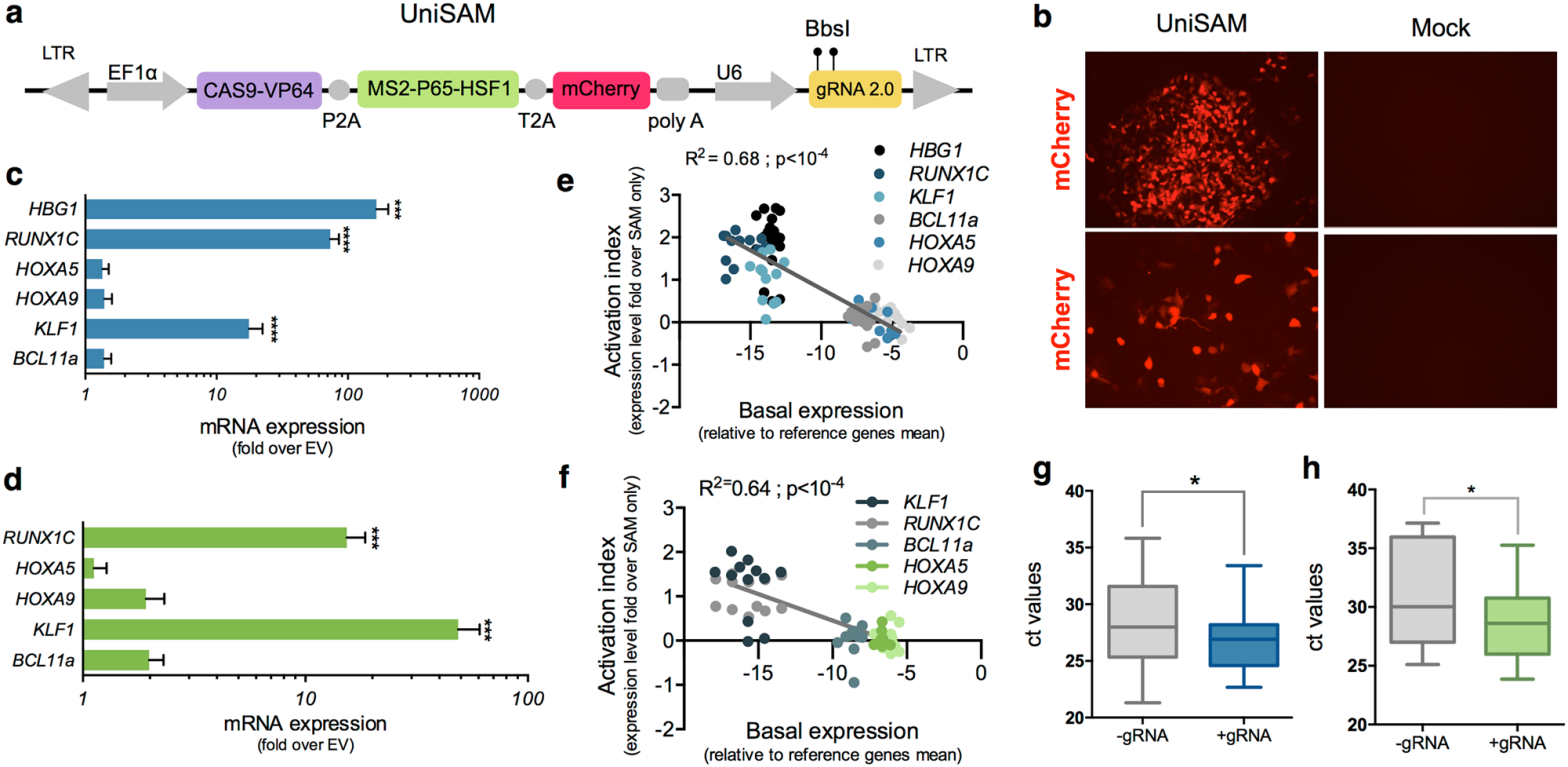
Design and assessment of all-in-one UniSAM vectors in HEK293T and HeLa cells. (**a**) Schematic of UniSAM vector in PiggyBac backbone. (**b**) Expression of mCherry in HEK293 and HeLa cells demonstrates efficient transfection and expression of the UniSAM vector. (**c**) Activation of five transcription factors and HBG1 following transfection of UniSAM vectors carrying single gRNAs, in HEK293T cells. (**d**) Activation of five transcription factors with single gRNAs, in HeLa cells. Data in (**c**) and (**d**) represent the mean activation by 4–6 single gRNAs for each gene (n = 3, Mann Whitney t-test). (**e**,**f**) Relationship between basal expression and activation levels for the different genes in HEK293T (**e**) and HeLa (**f**) (n = 72 from 3 independent experiments, linear regression with F-test). (**g**,**h**) Analyses of variance in Ct values following activation in HEK293T (**g**) and HeLa (**h**) (n = 3, F-test, p < 0.03).

The all-in-one vector (herein referred to as UniSAM) consists of the CAS9-VP64 and MS2-p65-HSF1 cDNAs separated by 2A peptides to ensure the generation of independent polypeptides (Fig. 1a). An mCherry tag located at the 3′ end of this cassette allows identification and/or isolation of cells that have been successfully transfected and that are expressing all preceding components (Fig. 1b). The cassette is under the control of the EF1α promoter and terminates with a synthetic polyadenylation signal. The vector also carries a U6 promoter driving the expression of the gRNA 2.0 backbone with a *BbsI* cloning site that enables cloning of the desired gRNA. This simple design means that activation plasmids for any gene of interest can be generate in a single step. All these components have been inserted into a PiggyBac backbone that can be used to mediate transient activation of gene expression or, in the presence of transposase, it can be integrated into the genome and subsequently excised allowing more precise temporal control of expression^11^. The smaller size of the PiggyBac vector allows for a lower total DNA to ORF ratio compared to lentiviruses, reducing the overall amount of DNA delivered and predictably increasing viability of transfected cells.

We generated a number of UniSAM vectors designed to activate genes encoding transcription factors involved in the production and differentiation of hematopoietic cells including *RUNX1c, HOXA9, HOXA5, KLF1 and BCL11a* ^12–15^. We designed 4-6 gRNAs for each gene using the online tools available at http://sam.genome-engineering.org/database/ (Supplementary Table S1), generated activation plasmids and tested their activity in HEK293 and HeLa cells (Fig. 1, Supplementary Fig. S1a). Two days after transfection significant activation of *RUNX1c* and *KLF1* expression was observed in both cell lines but there was minimal activation of *HOXA5, HOXA9 and BCL11a* (Fig. 1c,d). It was previously reported that inter-gene variability in transcriptional activation was linked to the basal expression^9^. Here we also show that gene activation was also inversely related to the basal expression level with *HOXA5, HOXA9 and BCL11a* being expressed at higher levels in both cell lines compared to *RUNX1c* and *KLF1* (Fig. 1e,f). Since gene activation mediates the recruitment of the transcriptional machinery we reasoned that genes that were being actively transcribed would be more resistant to further transcriptional activation and that there could be a limiting level of expression that this strategy would permit. To further test this hypothesis we compared all raw Ct values in the absence of gRNAs (empty vector, (EV)) with the value after the addition of gRNAs. We noted that the raw Ct values of all genes after activation were comparable to the overall basal levels of activation and that there was a reduction in the variance of their distribution (Fig. 1g,h). Taken together these data indicate that the level of mRNA expression achieved for any gene using this strategy avoids exceptionally high levels of gene expression.

**Figure 2:**
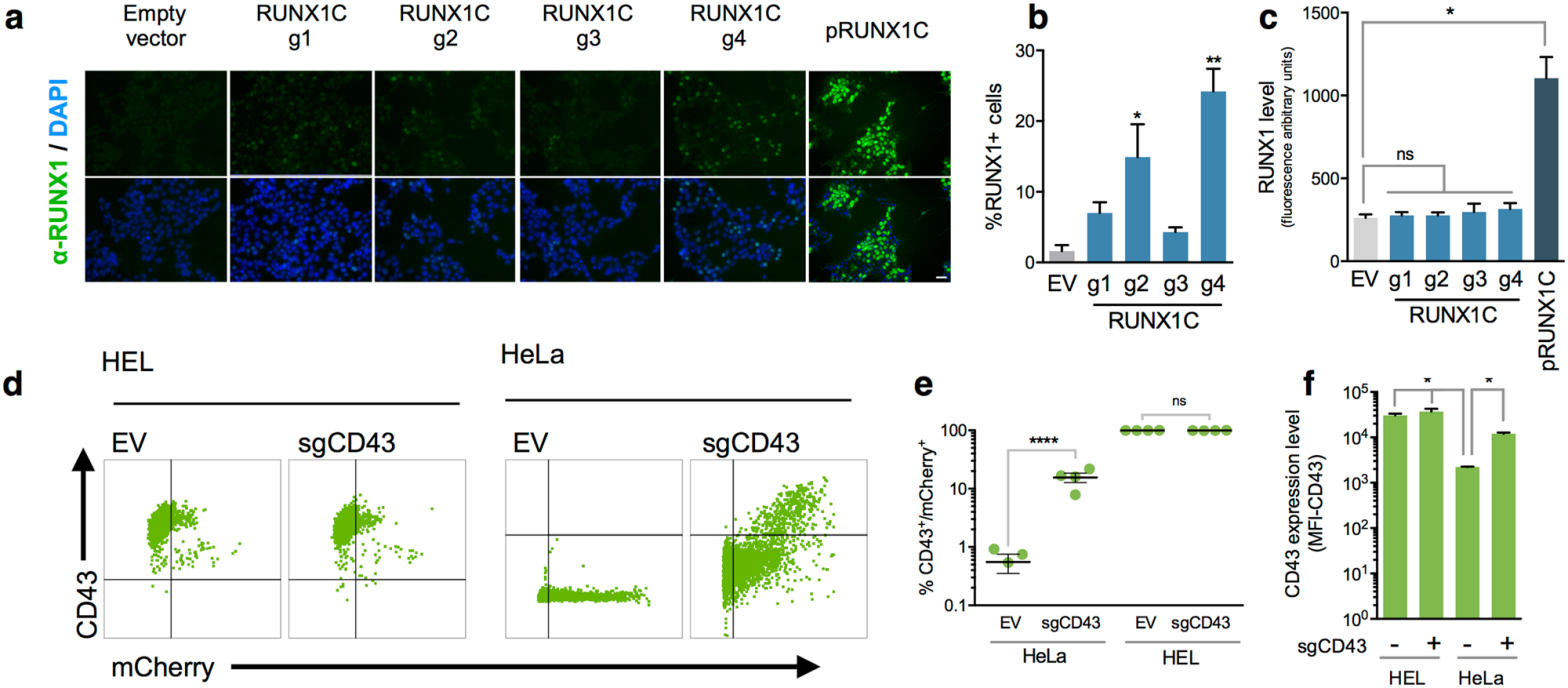
UniSAM gene activation results physiological levels of protein expression. (**a**) Immunocytochemistry analyses of HEK293T cells transfected with the UniSAM carrying no gRNA (empty vector (EV)) or a single gRNAs directed to RUNX1c (n = 4 for each of the gRNAs, g1 to g4) or a CMV-driven pRUNX1c expression plasmid (scale bar, 50 μm). (**b**) Percentage of RUNX1 + cells following transfection of UniSAM vectors carrying gRNA 1–4) (n = 4, Kruskal Wallis One-way ANOVA, *p < 0.05, **p < 0.01). (**c**) Comparison of RUNX1 expression levels in individual cells mediated by UniSAM and CMV-driven pRUNX1c expression plasmid analysed by Columbus Image Analysis Software (n = 4, Kruskal Walis One-way ANOVA, ns p > 0.5; *p < 0.03). (**d**) Flow cytometry analysis of HEL and HeLa cells transfected with the empty vector (EV) or a UniSAM containing a sgRNA for CD43. (**e**) Quantification of the proportion of CD43 + cells in (d) (n = 4; Sidak’s two-way Anova, ****p < 0.0001). (**f**) Quantification of CD43 expression level (assessed by mean fluorescent intensity (MFI)) in HEL and HeLa cells after activation (n = 4; Dunn’s one-way Anova; *p > 0.05).

To demonstrate that gene expression activation was also associated with increased protein production, HEK293T were transfected with the UniSAM-RUNX1c vectors and stained by immunocytochemistry. We observed an increase in the proportion of RUNX1^+^ cells, thus implying an increase in RUNX1c protein, and this varied depending on the gRNA used (Fig. 2a,b). RUNX1 protein was detected in a very small number of control, non-activated HEK293T cells and this allowed us to quantify the physiological level of protein expression in individual cells. Upon activation with the UniSAM system the level of protein expression within activated cells was comparable to the level in control cells but significantly lower than cells transfected with a standard CMV-driven RUNX1c expression plasmid (Fig. 2c). This further supports our hypothesis that there is an upper limit to the level of activation achieved using this strategy and that abnormally high, non-physiological levels, observed in classical transgenic approaches, are avoided.

To assess whether the UniSAM strategy could be used to alter the expression of a cell surface marker we activated *SPN* (which encodes for CD43) in HeLa cells and in human erythroleukaemia (HEL) cell line. CD43 is expressed in HEL but not HeLa cells so this system allowed us to test the influence of the basal expression on the level of activation and to assess the upper limits of gene activation at the protein level. We successfully activated the expression of CD43 on the cell surface of HeLa cells with a single gRNA for CD43 (Fig. 2d–f) but the addition of the same gRNAs to HEL cells, where CD43 was already expressed a relatively high level, had no significant effect (Fig. 2e,f). This experiment further confirms that the basal expression is a strong inferring factor in achieving activation and that the system avoids high non-physiological levels of expression.

**Figure 3:**
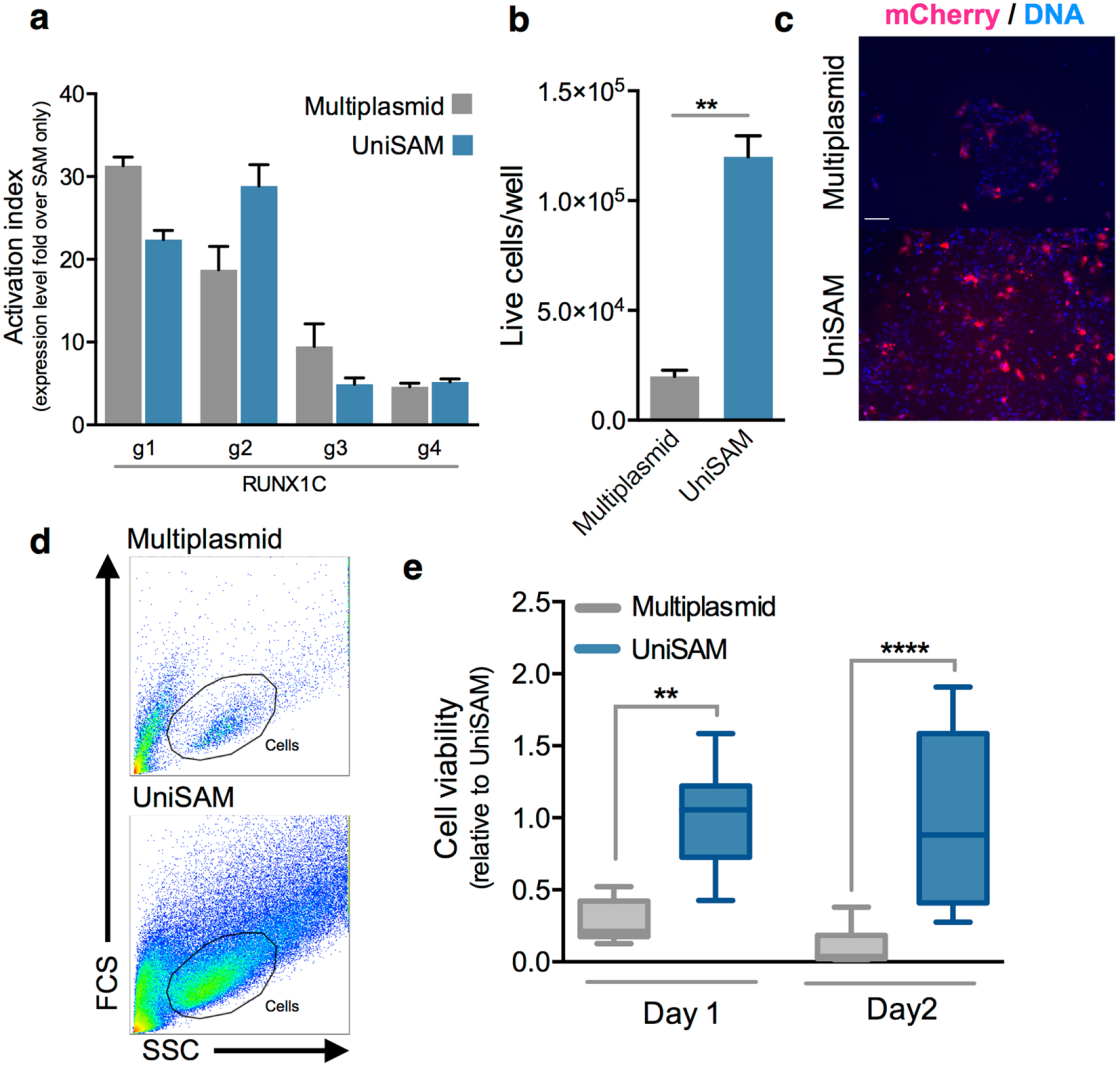
Direct comparison of the UniSAM system and the multiplasmid system. (**a**) Activation of RUNX1c using the UniSAM or multiplasmid systems with four different gRNAs (g1-g4) in HeLa (n = 3, Mann-Whitney t-Test). (**b**) Quantification of cell viability of hESCs one day after transfection as measured by Trypan Blue staining (n = 5; Mann-Whitney t-test, **p < 0.01). (**c**) Visual microscopic appearance of transfected hESCs (identified by mCherry expression) one day after transfection indicating that there are more live cells using the UniSAM system (scale bar indicates 50 μm). (**d**) Flow cytometry analysis of hESCs samples collected two days after transfection, gate indicates cells. (**e**) Comparison of the viable cells at day 1 and day 2 post transfection of hESCs, as measured by flow cytometry analyses of DAPI staining (n = 3; Dunn’s one-way Anova; **p > 0.01, ****p < 0.0001).

To assess whether our all-in-one UniSAM vector offered a significant advantage over the previously published multiplasmid strategies we compared the two systems directly. HeLa were transfected with equimolar quantities of the coding elements of the two plasmid systems including gRNAs to the *RUNX1c* locus then gene activation was assessed by qPCR. We demonstrated that the UniSAM and multiplasmid approach mediated comparable activation of RUNX1c for all four gRNAs (Fig. 3a). It is interesting to note that g2 seems to be the most efficient gRNA in Hela cells whereas in HEK293, g4 was the most efficient indicating a cell line depend effect of the specific gRNAs. When we directly compared the two strategies in hESCs we noted a significant difference in cell viability. RUNX1C-GFP hESC that were transfected with the multiplasmid system exhibited a significantly higher amount of cell death one day after transfection, when compared to the UniSAM strategy (Fig. 3b,c). This reduced cell viability was confirmed by flow cytometry both at one and two day after transfection (Fig. 3d,e). Thus, our unique UniSAM strategy is apparently able to mediate comparable gene activation levels compared to the published multiplasmid strategies but the significant improvement on cell viability makes it a more attractive strategy to use, particularly in cell types that are sensitive to transfection.

**Figure 4:**
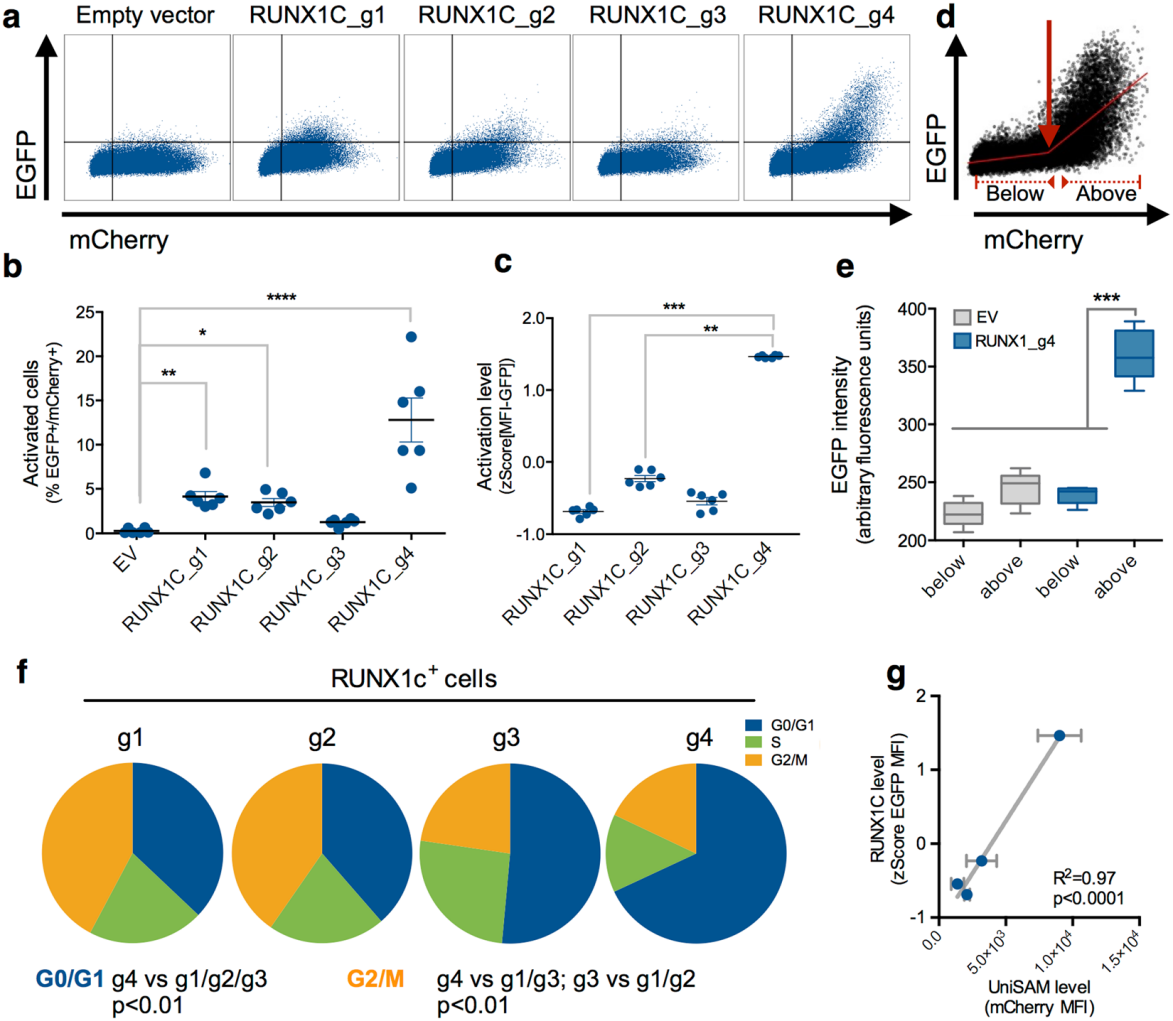
Functional assessment of UniSAM vectors in human pluripotent stem cells. (**a**) Flow cytometry analyses of RUNX1C-EGFP reporter hESCs transfected with UniSAM vectors carrying no gRNA (empty vector (EV)) or single gRNAs (g1-g4) directed to RUNX1C showing transfection efficiency (mCherry) and gene activation (EGFP) (n = 6). (**b**) Quantification of the number of activated cells as a proportion of transfected cells (n = 6, Kruskal Wallis One-way ANOVA, *p < 0.05, **p < 0.01, ****p < 0.0001). (**c**) Quantification of the level of gene activation by the different gRNAs indicated by the mean fluorescence intensity (MFI) of EGFP (n = 6, Kruskal Walis One-way ANOVA, **p < 0.01, ***p < 0.001). (**d**,**e**) ‘Breakpoint’ in the activation level mediated by RUNX1c_g4 (n = 6) and (quantification (**e**) of the fluorescence above and below that breakpoint (n = 6, one-way ANOVA, ***p < 0.001). (**f**) Proportion of RUNX1C-GFP-expressing cells in each stage of the cell cycle after activation by gRNAs g1 to g4 (n = 3, one way ANOVA). (**g**) Linear regression analyses of UniSAM expression level (mCherry) versus RUNX1c gene activation (EGFP) (n = 6; F-test).

This increased viability allowed us to assess the UniSAM strategy in the RUNX1C-EGFP reporter hESC line where we were able to assess the heterogeneity of activation between individual cells^14^. The transfection efficiency of UniSAM vectors was monitored using the mCherry reporter and the level of activation of the endogenous gene was assessed by expression of EGFP, knocked-in under the distal promoter that specifically drives RUNX1c^14^ (Fig. 4a). The presence of the different RUNX1c-gRNAs resulted in the emergence of varying proportions of RUNX1C-EGFP cells (Fig. 4b) and the different gRNAs mediated different levels of EGFP expression (Fig. 4c). The level of activation by the different gRNAs in hESCs showed a similar trend to that observed in HEK293 cells with g4 being the most powerful (Figs 1g, 4b–c). Flow cytometry allowed analysis at the single cell level and this identified a correlation between the level of UniSAM in each cell (reported by mCherry) and the level of gene activation (Fig. 4d) that was not possible using the less sensitive immunocytochemistry analyses. Statistical analysis of flow cytometry data for the RUNX1C-g4 uncovered a bimodal trend in expression of EGFP and piecewise regression identified a threshold of UniSAM expression that was consistent between experiments (Supplementary Figure S1b,c). As expected the activation level above that threshold ‘breakpoint’ was significantly higher than that below (Fig. 4e). These data show that the level of activation can be tunable by modifying the level of expression of the Cas9-SAM effector and/or the amount of gRNA in agreement with previously studies that have shown that altering the amount of gRNA can mediate the activation level^16^.

We observed that RUNX1C-EGFP was activated in a subpopulation of successfully transfected cells, comparable with that previously reported in hESCs using a drug-selectable dCas9-VP64 strategy^17^. Here we were able to test whether gene activation was occurring preferentially in cells at specific stages of the cell cycle. Flow cytometry analysis of Hoechst-stained cells revealed that activated cells (mCherry^+^EGFP^+^) were present in cells at all stages of the cell cycle when each of the 4 gRNAs was used (Fig. 4f). Interestingly, cells activated by g1 and g2 were slightly enriched for cycling cells (G2/M) whereas the majority of cells that were activated by g4 were in G0/G1. To determine whether this was a preferential susceptibly to activation at a particular stage or an effect of RUNX1 C activation itself we evaluated the mCherry intensity that would be diluted out as cells divide. There was an inverse correlation between the level of RUNX1C (EGFP) and mCherry, indicating that cells expressing high levels of RUNX1C were retaining mCherry expression suggesting a lower rate of proliferation in that population (Fig. 4g). Thus the higher proportion of cells in G0/G1 is more likely caused by the higher levels of RUNX1C rather than being an effect of preferential activation at this stage in the cell cycle.

The ultimate aim of this gene activation strategy in hESC would be to modify cell fate and/or to enhance *in vitro* differentiation protocols. In a proof of principle experiment to demonstrate that genes activated in this way could modify cell fate we tested the strategy in a well-established transdifferentiation assay that requires activation of a single gene^6, 18^. We designed three gRNAs predicted to activate murine *MyoD*1, generated UniSAM-MyoD1 vectors and integrated these into the genome of murine fibroblast using the HyPBase Transposase^7, 19^. A pMyoD1 vector was used as a positive control for fibroblast-myocyte transdifferentiation. In our preliminary experiments we observed increased expression of MyoD1 and the presence of multinucleated syncytium expressing Myosin Heavy Chain I (MHCI) protein in cells that had been transfected with the UniSAM-MyoD1_g3 vector but not in cells transfected with the control empty vector indicating that a proportion of fibroblasts had transdifferentiated into myocyte by activation of *MyoD1* (Supplementary Figure S1d,e).

In summary we have designed and generated a UniSAM vector that can be used to activate the expression of endogenous genes with the potential to modify cell fate without the need for drug selection. The strategy allows the direct comparison of cells in which genes have been activated with those that have not under the same experimental conditions and so provides a tool to understand the heterogeneity of activation at the single cell level. Importantly the increased cell viability using the UniSAM vector compared to multiplasmid strategies provides a system that can be used in transfection-sensitive cell lines. Thus the strategy can be exploited to finely tune the expression of transcription factor networks and thus to modulate the differentiation pathways of pluripotent stem cells in the quest to produce therapeutic cell types.

## Methods

### UniSAM vector production

The PiggyBac backbone was prepared by *NheI* and *PacI* digestion of the PB- CAG-hCD2 (Kind gift of Kesiure Kaji). EF1α-Cas9-Vp64 (Addgene-61422) was also digested with *NheI* and *PacI* (New England Biolab) and ligated into PiggyBac backbone to generate the PB-EF1α-Cas9-VP64. The P2A-MS2-p65-HSF1 and the T2A-mCherry-PolyA-U6-gRNA2.0 backbone were synthesized as double strand DNA (Thermo Scientific and Integrated DNA technology, respectively). All *BbsI* sites, except in the gRNA backbone were mutated and codon optimized according to the codon usage in Homo sapiens. Overlapping regions for Gibson assembly were included in the design of synthetic dsDNA fragments. Flanking SapI sites were added to release the synthetic dsDNA fragment following subcloning into pGEMT easy vector (Promega). PB-EF1α-Cas9-VP64 was linearized by *NheI* digestion and assembled at 50 °C for 1 hour with *Sap1*-digested P2A-MS2-p65-HSF1 and T2A-mCherry-PolyA-U6-gRNA2.0 backbone. Correctly assembled UniSAM vector was confirmed by complete Sanger sequencing. gRNAs were obtained from Integrated DNA technology as two single strand oligos and annealed in 20 μl reaction in Quick Ligation Buffer (Promega) with 9 μl of each oligos and annealedin in the thermal cycler 95 °C for 5 minutes fo llowed by cooling to 25 °C with 1 °C/minute ramp. 25 ng of purified *BbsI* linearized UniSAM or 25 ng of Purified *BsmBI* linearized lenti sgRNA(MS2)_puro (gift from Feng Zhang -Addgene plasmid #73797) was ligated with 1 μl of annealed gRNA diluted 1:500 using the Quick Ligation Kit (Promega). Ligation was used to transform Top10 *E.Coli*, correctly ligated gRNAs were confirmed by Sanger sequencing. UniSAM plasmid is available from Addgene (ID 99866).

### Cell culture and transfections

HEK293T were cultured in Glasgow Minimal Essential Medium (GMEM) with Non Essential Amino Acid, GlutaMax and 10% FCS (Gibco) and passaged every few days, at the ratio 1:6. HeLa cells were cultured in Dulbecco’s Modified Eagle Medium/Nutrient Mixture F-12 (DMEM/F12) with Glutmax and 5% FCS (Gibco) and passaged every few days, at a ratio of 1:6. HEL were cultured in Iscove’s Modified Dulbecco’s Medium (IMDM) with 10% FCS (Gibco) and passaged every few days, at a ratio of 1:4. 2 × 10^5^ cells were plated, transfected at 6–8 hours with 0.75 μg of DNA using Xfect Transfection reagent (Clontech) and then analysed 2 days after. Cells were also transfected with 0.5 μg CMV-RUNX1C (Addgene-12426) for the overexpression. RUNX1C-EGFP hESCs were cultured in StemPro hESC SFM (Gibco) with 20 ng/ml bFGF (R&D). Wells were coated with CELLstart at least 1 hour before plating and cells were passaged using the StemPro EZPassage tool (Thermo﻿Fisher Scientific). Media change was performed every day and cells passaged every 3–4 days at a ratio of 1:4. 3 × 10^5^ cells were plated into a coated 6 well plate and reverse transfected with 2 μg of UniSAM DNA using the Xfect Transfection reagent (Clontech) and analyzed 2 days later. For the comparison with the multiplasmid system, 0.5 μg UniSAM or equimolar ratio of the Multiplasmid and 1 μl of Lipofectamine 2000 reagent (ThermoScientifc) were used with pmCherryN1 (Clonetech) being co-transfected to monitor transfected cells by flow cytometry. The multiplasmid systems consisted of lenti sgRNA(MS2)_puro optimized backbone (Addgene plasmid #73797); EF1α-Cas9-Vp64-Blast (Addgene plasmid #61425); lenti MS2-P65-HSF1-Hygro (Addgene plasmid #61426). To achieve an equimolar ratio of all the parts of the multiplasmid vectors the mass ration of UniSAM:Cas9-VP64:MS2-p65-HSF1:sgRNA (1:1.14:0.95:0.68) was used.

Primary mouse embryonic fibroblasts (kindly provided by Kaji Keisuke) were cultured in GMEM with Glutamine/Pyruvate and 10% FCS (Gibco) on Gelatin-(Sigma Aldrich) coated flasks. 1.5 × 10^5^ fibroblasts, between passage 2 and 4, were plated overnight and then transfected with 1.5 μg of UniSAM and 0.5 μg of Transposase HyPBase^19^ or 1.5 μg of CMV-MyoD1 (gift of Andrea Corsinotti) using 6 μl of FuGENE Transfection Reagent (Promega). Two days after transfection fibroblast were maintained under starvation condition in GMEM, Glutamine/Pyruvate and 2% Horse serum (Sigma Aldrich) and then analysed by immunocytochemistry 7.5 days later.

### Gene expression

Total RNA was purified using the RNAeasy Mini Kit (Qiagen) and cDNA synthesized from 500 ng of total RNA using the High Capacity cDNA synthesis Kit (Applied Biosystem). 2 ng of cDNA were amplified per reaction and each reaction was performed in triplicate using the LightCycler 384 (Roche) with SYBR Green Master Mix II (Roche). A melting curve was performed and analyzed for each gene to ensure the specificity of the amplification. For human RNA expression analyses *GADPH*, *β-Actin* and *B2M* were used as reference genes and the geometrical mean was used to normalize the data. For mouse RNA expression analysis the *Sdha* reference gene was used to normalize the data. Primer sequence and efficiencies are reported in Supplementary Table 2.

### Immunocytochemistry

Cells were fixed in 4% PFA in PBS at room temperature for 10 minutes, permeabilized in PBS-T (Triton-X100 (0.2% for the MHCI and 0.4% for the RUNX1)) for 20 minutes and blocked in PBS-T with 1% BSA and 3% goat serum for 1 hour. Primary antibodies were incubated in blocking solution over night at 4 °C (RUNX1 1:200 - ab92336, Abcam) or room temperature for 2 hours (MHCI at 2 μg/ml, MF-20, DSHB). Cells were then washed in PBS-T and incubated with secondary antibodies for 1 hour at room temperature (for RUNX1: donkey α-rabbit 1:200 - A-11008; for MHCI goat α-mouse 1:1000- A11017 or goat α-mouse 1:1000- A10036 (Thermo Scientific). Cells were washed in PBS-T and counterstained with DAPI. Images were generated using the Zeiss Observer microscope. Specific algorithm was developed for the Operetta Imaging System to analyse the numbers of RUNX1 + cells and the staining intensity (Perkin Elmer). Cell nuclei were first identified using the DAPI staining with dimension and intensity cut offs being set to minimize inaccurate nuclear identification. Using control cells the RUNX1 + gate was then set to identify the nuclei expressing RUNX1 and the intensity of Alexa Fluor 488 staining was measured for each RUNX1-positive nuclei.

### Flow cytometry

Single cell suspensions were obtained by Trypsin treatment (Gibco), resuspended in PBS with 1%BSA and 5 mM EDTA and analysed in the LSR Fortessa Analyser (BD). Dead cells were gated out using DAPI staining. For the cell cycle analysis cells were stained with Hoechst 33342 at the final concentration of 10 μg/ml in the culture medium 2 hours prior to harvesting. Cells were kept on ice until analysed using the LSR Fortessa Analyser (BD). For cell viability comparison between the UniSAM and the multiplasmid system, cells were acquired for 90 seconds at a medium speed to acquire the same volume of cell solution to ensure comparability in cell viability between samples. For CD43 cell surface staining, single cell suspension of HeLa and HEL cells were obtained by StemPro Accutase Cell Dissociation Reagent (Gibco), washed, blocked in PBS with 1% BSA and resuspended in staining solution (PBS with 1% BSA) with mouse anti-human CD43-APC (1:100)(eBioscience) for 10 minutes at room temperature before being washed and analysed using the LSR Fortessa Analyser (BD). FlowJo 10.1 was used for all flow cytometry data analyse.

### Statistical and R analyses

Results are expressed as mean ± standard error mean. Statistical analyses were performed using Graph Pad Prism 6.0 with the exception of segmented fitting (Fig. 2d and Figure S1b) performed with R. Identification of the breakpoints has been performed using the R package “segmented”.

## Electronic supplementary material

Supplementary Information
