# Supplementary material for: An all-in-one UniSam vector system for efficient gene activation
Source: Sci Rep. 2017 Jul 25;7:6394. doi: 10.1038/s41598-017-06468-6 (PMC5526871; doi:10.1038/s41598-017-06468-6)
Supplement: Supplementary file 1 — Supplementary Information [file 41598_2017_6468_MOESM1_ESM.pdf]

An all-in-one UniSAM vector system for efficient gene activation

Antonella Fidanza[1,*], Martha Lopez-Yrigoyen[1], Nicola Romanò[2], Rhiannon Jones[1], A Helen Taylor[1], Lesley M Forrester[1,*].

1. Centre for Regenerative Medicine, University of Edinburgh, Edinburgh, UK
2. Centre for Integrative Physiology, University of Edinburgh, Edinburgh, UK

* Correspondence to afidanza@ed.ac.uk, L.Forrester@ed.ac.uk

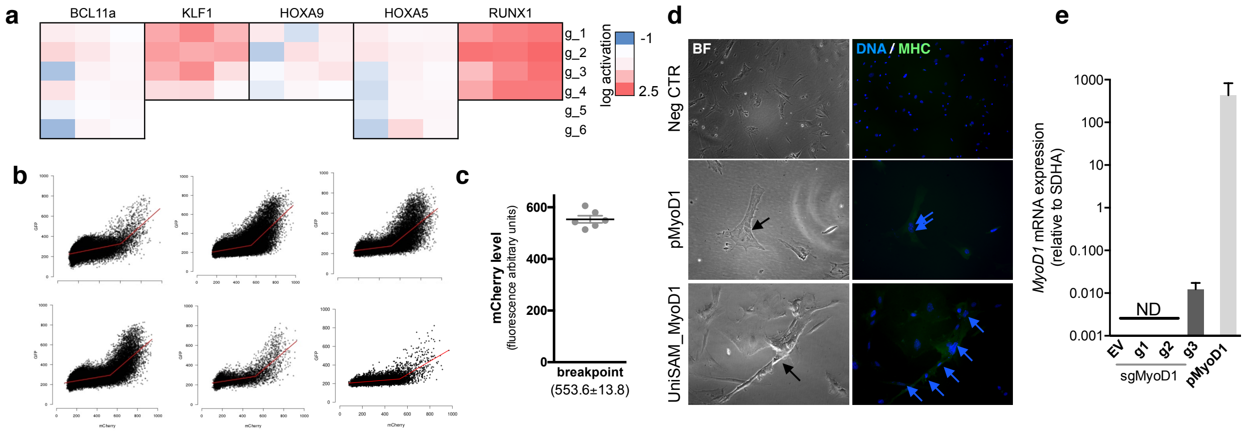

Supplementary Figure S1

(**a**) Heatmap of levels of gene activation (log scale) observed by the different single gRNAs directed to the five different transcription factors. (**b**) Flow cytometry analyses of RUNX1C-GFP hESCs after transfection with UniSAM RUNX1_g4 in six independent experiments demonstrating the consistency of the threshold “breakpoint’ between experiments despite difference in transfection efficiencies. (**c**) Quantification of threshold ‘breakpoint’ of mCherry expression demonstrating the level of transfection required for gene activation. (d) Bright field (BF) (left) and immunocytochemistry analyses using an anti-MHC antibody (right) of mouse embryonic fibroblasts transfected with a UniSAM empty vector carrying no gRNA (Neg CTR) or a gRNA directed to MYOD1 (UniSAM_MyoD1_g3). The presence of syncytia of Myosin Heavy Chain 1 (MHC)-expressing myocytes indicates transdifferentiation by the UniSAM_Myod1 vector and a control vector carrying the MyoD1 cDNA (pMyoD). Blue arrows point to nuclei in multinucleated MHC1+ myotube. (e) Gene expression analysis show MyoD1 expression in MEFs trisected with sgMyoD1_g3.

gRNA name	gRNA sequence	TSS distance	DNA strand
RUNX1C_g1	GGGCAGGTGGAGGGAAGGAA	126	b
RUNX1C_g2	TTTCTTGCACAGCCTGGGGG	147	b
RUNX1C_g3	TTGAGATGGGCTGTGGAAAG	40	b
RUNX1C_g4	CATCACTTAAGTCACATGAT	73	b
KLF1_g1	CCTCTCTGTCCTTAGCTGAT	25	t
KLF1_g2	CGGCGGGGGGGCACTGTTTC	81	t
KLF1_g5	AAACTTCACGTTGGCCTGTC	109	t
KLF1_g6	TTGACTTGGCTTTGGACACA	154	b
KLF1_g7	GGCTGTGATAGCCCCTTCGA	0	t
HOXA5_g1	AGGGGAGTTGGGTGGAGGCG	159	t
HOXA5_g2	GTGCACGAGTTTACCTCTAG	135	b
HOXA5_g3	CATCAGGCAGGATTTACGAC	111	b
HOXA5_g4	TACTTGGTTCCCTCCTACGT	41	t
HOXA5_g5	CGAAGTCGTACCCCATATTT	69	b
HOXA5_g6	TGATGAATTATGGAAATGAC	12	t
HOXA9_g1	GCCGGCGCCCGCGCCCCCAT	39	t
HOXA9_g2	GGACGGGCACGTGACGCGCA	65	t
HOXA9_g4	ATCGGACCATTAATAGCGTG	114	b
HOXA9_g5	TTTACGCGTTATTGTTCTGC	86	b
BCLL1a_g1	AGAGAGAGAGATGAAAAAAA	49	t
BCLL1a_g2	GAGAGAGAGAAGAGAGATAG	19	t
BCLL1a_g3	GGCAGGGCGAGCAGGAGAGA	168	t
BCLL1a_g4	CTTGAACTTGCAGCTCAGGG	81	b
BCLL1a_g5	GGACAGAGACACACAAAACA	146	t
BCLL1a_g6	TCCCTGCGAACTTGAACGTC	114	t
HBG1_g1	TGAGGCCAGGGGCCGGCGGC	46	b
HBG1_g2	GCTATTGGTCAAGGCAAGGC	107	t
HBG1_g3	GGCTAGGGATGAAGAATAAA	25	b
HBG1_g4	GCTAAACTCCACCCATGGGT	133	b
HBG1_g5	TGGTCAAGTTTGCCTTGTCA	85	t
HBG1_g6	TATCTGTCTGAAACGGTCCC	155	b
CD43_g	GAAGCGTGGGATCTGGAATC	151	b
Myod1_g1	GGGCGGAGCTTGGGGTCCCC	107	b
Myod1_g2	CCTGGCCCCAGTGGCTACCC	128	t
Myod1_g3	GATAAATAGCCCAGGGCGCC	11	t

Supplementary Table 1 : gRNA sequences, location expressed as the upstream distance to the transcriptional start site (TSS) and target DNA strand, bottom (b) or top (t).

Gene name	Accession Number	Forward	Reverse	Efficiency
B2M	NM_004048_2	TTCTGGCCTGGAGGCTATC	TCAGGAAATTTGACTTTCCATTC	2.07
GADPH	NM_002046_3	AGCCACATCGCTCAGACAC	GCCCAATACGACCAAATCC	2.05
β-ACTIN	NM_001101_3	CCAACCGCGAGAAGATGA	CCAGAGGCGTACAGGGATAG	2.06
RUNX1C	NM_001754.4	AGCCTGGCAGTGTCAGAAGT	GGGACTCAATGATTTCTTTTACCA	1.98
KLF1	NM_006563.3	ACACCAAGAGCTCCCACCT	GTAGTGGCGGGTCAGCTC	1.90
HOXA5	NM_019102.3	GCGCAAGCTGCACATAAG	CGGTTGAAGTGGAACTCCTT	1.913
HOXA9	NM_152739.3	CCCCATCGATCCCAATAA	CACCGCTTTTTCCGAGTG	2.00
BCL11A	NM_138559.1	CCAAACAGGAACACATAGCAGA	GAGCTCCATGTGCAGAACG	1.90
HBG1	NM_000559	TGGATCCTGAGAACTTCAAGC	GCCACTGCAGTCACCATCT	2.10
Sdha	NM_023281.1	TGTTCAGTTCCACCCCACA	TCTCCACGACACCCTTCTG	2.00
MyoD1	NM_010866.2	AGCACTACAGTGGCGACTCA	GGCCGCTGTAATCCATC	1.96

Supplementary Table 2 : Primer sequences, accession number and efficiency calculated using the LightCycler 480 Software version 1.5.
